# Liquid biopsies for early diagnosis of brain tumours: *in* *silico* mathematical biomarker modelling

**DOI:** 10.1098/rsif.2022.0180

**Published:** 2022-08-03

**Authors:** Johanna A. Blee, Xia Liu, Abigail J. Harland, Kavi Fatania, Stuart Currie, Kathreena M. Kurian, Sabine Hauert

**Affiliations:** ^1^ Department of Engineering Mathematics, University of Bristol, Ada Lovelace Building, Bristol BS8 1TW, UK; ^2^ Brain Tumour Research Centre, Bristol Medical School, Bristol BS2 8DZ, UK; ^3^ Department of Radiology, Leeds General Infirmary, Great George Street, Leeds LS1 3EX, UK

**Keywords:** glioblastoma multi-forme (GBM), mathematical modelling, biomarkers, glial fibrillary acidic protein (GFAP), brain tumours, cancer

## Abstract

Brain tumours are the biggest cancer killer in those under 40 and reduce life expectancy more than any other cancer. Blood-based liquid biopsies may aid early diagnosis, prediction and prognosis for brain tumours. It remains unclear whether known blood-based biomarkers, such as glial fibrillary acidic protein (GFAP), have the required sensitivity and selectivity. We have developed a novel *in* *silico* model which can be used to assess and compare blood-based liquid biopsies. We focused on GFAP, a putative biomarker for astrocytic tumours and glioblastoma multi-formes (GBMs). *In silico* modelling was paired with experimental measurement of cell GFAP concentrations and used to predict the tumour volumes and identify key parameters which limit detection. The average GBM volumes of 449 patients at Leeds Teaching Hospitals NHS Trust were also measured and used as a benchmark. Our model predicts that the currently proposed GFAP threshold of 0.12 ng ml^−1^ may not be suitable for early detection of GBMs, but that lower thresholds may be used. We found that the levels of GFAP in the blood are related to tumour characteristics, such as vasculature damage and rate of necrosis, which are biological markers of tumour aggressiveness. We also demonstrate how these models could be used to provide clinical insight.

## 1. Introduction

Gliomas are the largest group of intrinsic brain tumours, with age-adjusted incidence rates ranging from 4.67 to 5.73 per 100 000 [1]. Furthermore, malignant gliomas cause significant years of life lost compared with other cancer types—about 20 years of life lost on average—due to late diagnosis and poor treatment outcomes [2]. Currently, brain tumours are diagnosed and assessed using scans (e.g. magnetic resonance imaging (MRI) and computed tomography (CT)), histology and molecular profiling. A blood-based liquid biopsy could provide a cheap, simple and minimally invasive way to diagnose brain tumours and monitor for recurrence.

Astrocytomas are the most common type of glioma. One biomarker for astrocytic tumours is glial fibrillary acidic protein (GFAP) [3–5], which is an intermediate filament protein present in astrocytes that is not found outside of the central nervous system (CNS). The normal blood–brain barrier (BBB) is comprised of endothelial cells and astrocytes which tightly restrict the transportation of GFAP into the blood [6]. GFAP can be used as a blood-based biomarker of neurological disease as its presence in the blood is indicative of astrocyte injury or necrosis as well as BBB damage [7,8]. The most common and highest grade of astrocytoma is glioblastoma multi-forme (GBM). GBMs are associated with astrocyte necrosis and BBB breakdown which could allow GFAP to enter the bloodstream. It is currently unknown exactly how the levels of serum GFAP relate to glioma properties and whether current methods are sensitive or selective enough to use GFAP as a blood-based biomarker for astrocytic tumours.

Prospective studies have investigated using GFAP as a blood-based biomarker for astrocytomas. Serum GFAP was not detected above the analytical sensitivity of 0.08 ng ml^−1^ in most astrocytomas (74%), but was detected in most GBMs (89%) [9]. Receiver operating characteristic curve (ROC) analysis gave a GFAP serum cut-off limit of 0.12 ng ml^−1^ for optimized detection of GBMs [5,9]. Although the GFAP serum concentrations of most astrocytomas are far below this limit, some astrocytomas exceed this limit with GFAP levels of up to 2.04 ng ml^−1^ [9]. Some larger GBMs far exceed this limit (median 0.38 ng ml^−1^ reaching 11.4 ng ml^−1^) [9]. A lower detection threshold limit of 0.08 ng ml^−1^ has been suggested for distinguishing all grades of glioblastomas from healthy individuals [9].

Serum GFAP has been linked to prognosis, with GFAP greater than 0.2 ng ml^−1^ associated with significantly lower survival-free prognosis [9]. Serum GFAP levels are also associated with prognostic markers and progression-free survival [3]. They also correlate with tumour volume [3,5,9,10]. It has therefore been suggested that GFAP may be used in a wide range of different diagnosis and monitoring situations for astrocytic tumours. However, GFAP concentrations are extremely heterogenous across different tumours; for example, small tumours have been observed with very large serum GFAP concentrations and vice versa [3,9–11].

Brain tumour growth is complex and heterogenous, and it remains difficult to quantify. A lack of *in vivo* data, especially from the earlier stages of tumour growth, makes quantification challenging. To overcome this, a range of experimental and mathematical modelling techniques have been used to understand tumour growth dynamics [12–15]. Several models of glial tumour growth have been proposed [12,15]. One of the simplest models is exponential growth, in which it is assumed that the tumour has a constant volume doubling time [16]. There have been various adaptions to this original exponential model and there are several related models, such as linear growth models [12]. However, it has been shown that brain tumours, including GBMs, reach a plateau phase and are better represented by Gompertzian growth [14,17,18]. The Gompertzian growth model assumes an initial exponential phase, followed by a linear phase and finally a plateau phase. It is one of the most acknowledged models for tumour growth [19–21] and the one used in this study.

Mathematical modelling offers a powerful way of scanning a wide range of different scenarios in a time- and cost-efficient way. It can also offer additional mechanistic understanding and in the future may be used alongside current clinical methods to guide more effective treatment strategies (e.g. [19,22,23]).

Mathematical modelling has been used to explore the use of blood-based biomarkers for several different cancers [24–26]. However, no models have been developed for blood-based biomarkers for brain tumours. The key difference between modelling blood-based biomarkers in the brain and other tissues is the presence of the highly selective BBB. In the previous models of other tissues, it has been assumed that the fraction of the biomarker which enters the blood is constant and does not depend on tumour growth [24,25]. This assumption is not valid for the brain, as biomarkers from the brain are only able to enter the blood in significant quantities when the BBB is compromised. We therefore developed a new set of models to fulfil this unique requirement of the brain.

**Figure 1.**
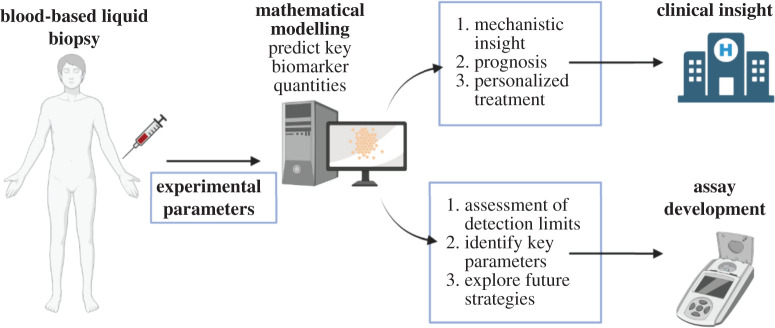
Concept of how mathematical modelling may be used in the development and deployment of blood-based biomarkers for brain tumours. This figure summarizes the role mathematical modelling could play both at the assay development stage and in clinical implementation.

We developed mathematical models which describe dynamic serum biomarker kinetics in relation to brain tumour growth. In this study, we focused on the CNS-specific intermediate filament protein GFAP [27,28]. We investigate current detection limits, show how the serum GFAP levels depend on the tumour characteristics, determine the key parameters which limit detection and explore future strategies. Computational modelling may be used to simulate the key aspects of the detection and properties of blood-based biomarkers and streamline the process of liquid biopsy development [29–31]. Our results demonstrate how mathematical modelling may be used both at the developmental stage of liquid biopsies for brain tumours and in their interpretation. We also show how these models may be used to gain mechanistic understanding and provide clinical insight (figure 1). A generalized form of our model is presented, and the key components and framework are discussed so that it can be easily adapted for biomarkers produced by other mechanisms for brain tumours with varying properties.

## 2. Material and methods

### 2.1. Measurement of current glioblastoma multi-forme detection volumes

The neuro-oncology multi-disciplinary meeting records at Leeds Teaching Hospitals NHS Trust between 2014 and 2020 were retrospectively reviewed, and all adult patients (16 years and over) with histologically proven GBMs were included (*n* = 483). The average volume at which these GBMs were diagnosed was calculated. Formal ethics approval was granted under the project and license—enhancing understanding and prediction of cancer outcomes with baseline characteristics from routinely collected data (IRAS ID. 277122). Baseline data included patient age, sex and WHO performance status (see electronic supplementary material, methods).

Pre-operative imaging protocols varied, but typically consisted of T2 and T2-FLAIR (fluid attenuated inversion recovery), diffusion weighted, T1 pre- and post-gadolinium sequences (Gd-T1) and a volumetric T1-weighted sequence post-gadolinium. Enhancing tumour volume was estimated following the established protocol for GBMs which is most effective for their irregular shapes [32]. Orthogonal measurements in axial and craniocaudal axes, measured on axial and coronal Gd-T1 images, or multi-planar reconstructions of a volumetric Gd-T1 sequence, using the institutional picture archiving and communication system (PACS, Impax v. 6.5.3.3009, Agfa Healthcare, Mortsel, Belgium) with electronic callipers on a submillimetre (mm) scale. Three orthogonal measurements were multiplied and divided by two to estimate volume. The axial image with the largest tumour was identified, and two maximum perpendicular dimensions were measured. Using reformatted sagittal or coronal images, the maximum dimension in the craniocaudal axis was measured.

### 2.2. *In vitro* measurement of average glial fibrillary acidic protein per cell

We measured the average GFAP of human glioma stem cells (G144 GSCs) and human GBM cells (U251). To quantify the average GFAP per cell, we used a standard protocol to quantify GFAP expression using western blotting and protein quantification as described by Wang *et al*. [33].

The G144 GSCs were gifted by Dr Steve Pollard (University of Edinburgh) and cultured as monolayers in serum-free basal media (Sigma, D8437) with added supplements (see electronic supplementary material, methods, for full media recipe). The U251 commercial human GBM cells (Sigma, 09063001) were cultured in Minimal Essential Media (Gibco, 11095080) supplemented with 10% fetal bovine serum (Gibco, 10500-064) and 1% MEM non-essential amino acid solution (Gibco, 11140035). Both G144 and U251 were cultured at 37°C in 5% CO_2_.

Experiments were repeated for three different passages of each cell line and all presented errors are standard errors. Cells were pelleted per passage and each pellet was pipetted vigorously with 100 µl of lysis buffer (full details of buffer preparation can be found in the electronic supplementary material, methods). The lysate was incubated on ice for 15 min in 1.5 ml microtubes (Starlab, E1415-2210) before being centrifuged using a VWR MICROSTAR 17R at 4°C and 17 000 × *g* for 3 min. The supernatant was transferred to fresh microtubes and stored at –20°C.

The protein concentrations of the lysates were determined relative to eight known concentrations of bovine serum albumin (Thermo Scientific, 23209) using the Pierce™ bicinchoninic acid protein assay kit (Thermo Scientific, 23227), following the 96-well plate protocol. The absorbance at 570 nm was measured using an iMark™ Microplate Reader (BioRad, UK) and the accompanying Microplate Manager^®^ software.

The protein quantities of samples were determined using western blotting. A standard curve was created (electronic supplementary material, figure S1) and used to determine the protein content of the lysate. This was then combined with the average number of cells in the lysate to calculate the quantity of GFAP per cell. Full details of the western blot protocol can be found in the electronic supplementary material, methods, but briefly lysate samples were run alongside serial dilutions of protein standard (Recombinant mouse GFAP protein, abcam, ab226309) with a molecular weight protein ladder at each end (Biorad Precision Plus Protein Dual Color Standard, 1610374). The gel was placed under a voltage of 150 V for 1 h to separate the proteins using PowerPac™ basic power supply (BioRad).

The protein was transferred to a membrane and blocking was carried out. Membranes were then immunoblotted with the following antibodies: GFAP Polyclonal Rabbit antibody (1 : 2000, Dako, Z0334) and *α* Tubulin (1 : 5000, Millipore, DM1A). After incubation with specific secondary antibodies conjugated to peroxidase (Sigma) proteins were visualized by Clarity ECL substrate (BioRad) using the BioRad Chemidoc XRS + system and analysed using Image Lab software (BioRad). Electronic supplementary material, figure S2, shows an example of a western blot used for GFAP quantification. The intensity of the bands, which are proportional to protein levels, was quantified using ImageJ software.

### 2.3. *In silico* model of serum biomarker kinetics for brain tumours

**Figure 2.**
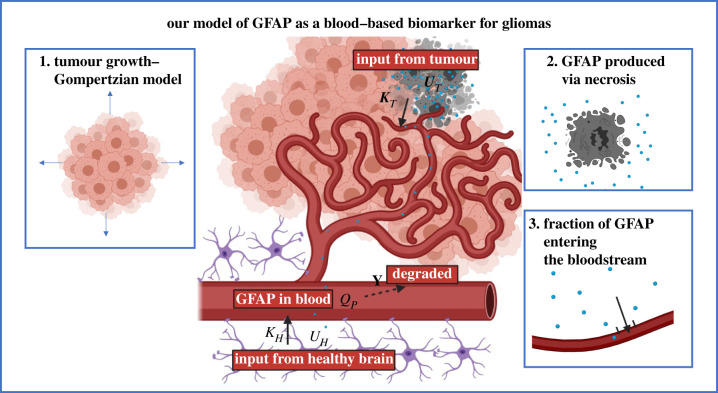
Our *in silico* dynamic model of serum GFAP with GBM growth. Schematic showing the key components of the model, with the input from the tumour (*K_T_*(*t*)*U_T_*(*t*)) and healthy tissue (*K_H_U_H_*) and the degradation rate (*γ*). The three key components of our model for GFAP are also highlighted: tumour growth, GFAP production via necrosis and the fraction of GFAP that can enter the bloodstream.

We developed a simple *in silico* model for brain tumour serum biomarker kinetics with three main components (figure 2). First, the model for tumour growth, second the mechanism via which the biomarker is produced and finally the process by which the biomarker enters the bloodstream (via breakdown of the BBB). This model was based on a compartmental model where biomarker kinetics are given by a set of differential equations. These were solved in MATLAB (MathWorks) using the ode45 ordinary differential equation solver.

In our model, it was assumed that the biomarker is well-mixed and homogenous in different compartments. We note that this does not fully encapsulate the heterogeneity of tumour growth but is a good first approximation that allows key parameters that influence the levels of biomarker in the blood to be explored.

The levels of biomarker in the blood were assumed to depend on the input from the tumour (*K_T_*(*t*)*U_T_*(*t*)) and healthy tissue (*K_H_U_H_*), as well as the rate at which the biomarker is degraded (*γ*). The mass of biomarker in the blood (*Q_p_*) is given by

2.1 $$\frac{dQ_{p}}{dt}=K_{T}(t)U_{T}(t)+K_{H}U_{H}-\gamma Q_{P},$$

where *U_T_*, *U_H_* are the tumour and healthy tissue production rates at time (*t*) and *K_T_*, *K_H_* are the corresponding fractions entering the blood. The concentration of biomarker in the blood is then equal to $\frac{Q_{p}}{V_{p}}$, where *V_p_* is the volume of blood. The serum GFAP concentration of healthy individuals (*C_H_*) is given by $C_{H}=\frac{K_{H}U_{H}}{\gamma }$. This model may be adapted for different biomarkers and applied to brain tumours with varying properties.

A range of growth models have been proposed to describe tumour growth [12,15]. Including simple exponential and linear models [12,16], however, GBMs reach a plateau phase and have been shown to be are better represented by Gompertzian growth [14,17,18]. We therefore employ a Gompertzian growth model:

2.2 $$V_{T}(t)=V_{T\max}\;\exp\left(\log\left(\frac{V_{T0}}{V_{T\max}}\right)\exp(-R_{T}t)\right),$$

where *V_T_*(*t*) is the volume of the tumour at time (*t*). To model the growth of necrosis, we assume that necrosis occurs after a specific tumour onset volume (*Vn*0) is reached. This volume corresponds to the onset time *Tn*0. We assume that necrosis also follows Gompertzian growth [13] so that the number of necrotic cells (*V_N_*(*t*)) is given by

2.3 $$V_{N}(t)=\{\begin{array}{c} \begin{array}{cc} 0,\; & t<Tn0\;\\ V_{n\max}\;\exp\left(\log\left(\frac{V_{N0}}{V_{N\max}}\right)\exp(R_{N}\;(t-Tn0))\right),\; & t\geq Tn0,\; \end{array}\; \end{array}$$

where *V_T_*_max_, *V_n_*_max_ are the tumour and necrotic plateau volumes, *V_T_*_0_ is the initial volume of the tumour (in this case taken as the volume of a single cell) and *R_N_*, *R_T_* are the initial growth rates. These volumes can be converted into corresponding numbers of cells using the average cellular densities. This Gompertzian growth model has been shown to be a good approximation of brain tumour growth [14,19,20]. However, it may be exchanged for any relevant model of brain tumour growth.

### 2.4. *In silico* model for glial fibrillary acidic protein as a blood-based biomarker

In this study, we focus on the CNS-specific intermediate filament protein GFAP. The key components of our dynamic model for GFAP are tumour and necrotic growth, biomarker production and the fraction entering the blood (figure 2).

Necrosis is a hallmark of tumour progression in astrocytic tumours. During necrosis, GFAP is released into the interstitial fluid (IF) [28]. GFAP has been found in the blood of patients with non-astrocytic tumours which suggests that GFAP may also be produced by other mechanisms [5,9,10]. For example, one patient with a cerebellar primarily diffuse large B-cell lymphoma was found to have very high serum GFAP levels (0.25 ng ml^−1^), possibly due to secondary tissue necrosis. However, for astrocytic tumours, it is generally accepted that GFAP is primarily produced via tumour necrosis [34–36] and so this is our model assumption (equation (2.4)). We also go on to then explore scenarios where this assumption might not hold, for example, when GFAP is produced via damage induced by the tumour to the surrounding brain.

For a tumour with GFAP produced via necrosis, the tumour production term ((*U_T_*(*t*)) would take the form

2.4 $$U_{T}(t)=\;Q_{N}\frac{dN_{N}(t)}{dt}=\{\begin{array}{cc} 0,\; & t<Tn0\;\\ Q_{N}R_{N}\log\left(\frac{N_{n\max}}{N_{N}(t)}\right)N_{N}(t),\; & t\geq Tn0,\; \end{array}$$

where *Q_N_* is the average mass of GFAP per dying cell, *N_N_* is the number of necrotic cells which is related the necrotic volume (*V_N_*) and the average necrotic cell density (*δ_N_*) by *N_N_* = *V_N_δ_N_*, *N_n_*_max_ is the maximum number of necrotic cells which is given by *N_n_*_max_ = *V_n_*_max_*δ_N_* and *Tn*0 is the time necrosis starts in the tumour.

After GFAP has been emitted into the IF, it may enter the blood either via BBB disruption [7] or via the glymphatic system [37]. The fraction of biomarker produced by the tumour which enters the blood (*K_T_*) is dependent on anything which affects the transport of biomarker into the bloodstream, as well as the removal or decay of the biomarker from the tumour. The breakdown of the glymphatic system, which is usually responsible for the rapid clearance of GFAP from the brain, may lead to enhanced retention of GFAP in the tumour [38,39]. This GFAP may then enter the bloodstream at a higher rate due to the increased permeability of the BBB [8]. Therefore, as the tumour grows, the fraction of GFAP which enters the bloodstream increases. We assume that these increases occur after a threshold tumour volume/time (*Vk*0/*Tk*0) is reached and can be approximated as a Hill function, so that *K_T_*(*t*) is given by

2.5 $$K_{T}(t)=\{\begin{array}{cc} K_{\min},\; & t<Tk0\;\\ K_{\min}-\frac{K_{\max}t^{h}}{K_{1/2}+t^{h}},\; & t\geq Tk0,\; \end{array}$$

where *K*_min_ and *K*_max_ are the minimum and maximum *K_T_* fractions, $K_{1/2}$ is the half-time constant, *h* is the Hill coefficient and *Tk*0 is the time at which the BBB is first compromised. We assume that when the BBB is intact (prior to tumour growth), the fraction of GFAP which enters the blood is zero (*K*_min_ = 0) and that $K_{1/2}>Tk0$. *K_T_* and *U_T_* are related, as necrosis occurs due to hypoxia, which is associated with blood vessel deformation. We therefore assume that changes in *K_T_* begin at the same time as necrosis starts so that *Tn*0 = *Tk*0.

If we simulate the dynamic serum GFAP as a function of time or tumour volume for a given set of parameters in our model, then we can obtain the corresponding tumour detection time (*t_d_*) and detection volume (*V_d_*). These define the time and volume at which the serum GFAP crosses the GFAP cut-off threshold limit used to identify GBM patients and so give the time and volume at which a tumour would be detected at the current threshold. If the parameters in our model change, the detection time and volume will also change. We define this detection volume change as $\Delta V_{d}=|V_{2d}-V_{1d}|$ where *V*_2*d*_ is the detection volume for the second set of parameters and *V*_1*d*_ is the detection volume for the original set of parameters.

### 2.5. Model parametrization and sensitivity analysis

Our model is governed by a set of parameters which define the serum GFAP in healthy patients, tumour growth, GFAP expression, necrosis and the entry of GFAP to the blood (due to a breakdown of the BBB). This model may be used to obtain a profile for a specific tumour based on its parameters, or to predict global averages based on average parameters for GBMs. We parametrize our model based on the experimental data available. For each parameter, we use the current experimental data to obtain global averages as well as ranges observed across GBMs (table 1). We use these to model the average behaviour as well as the impact of heterogeneity.

**Table 1.** Parameters for our *in silico* model of serum GFAP for an average GBM.

parameter	definition	average	range	reference
*R_T_*	initial rate of tumour growth	0.008 cells day^−1^	0.004–0.01 cells day^−1^	[40]
*R_N_*	initial rate of necrotic growth	0.009 cells day^−1^	0.005–0.01 cells day^−1^	[40]
*V_T_*_max_	tumour growth plateau constant	158 ml	72–164 ml	[14,18]
*V_N_*_max_	necrotic growth plateau constant	150 ml	70–159 ml	[14,18]
*Vn*0	volume of necrosis onset	0.5 ml	0.1–20 ml	[40,41]
*Vk*0	volume of *k* fraction onset	0.5 ml	0.1–20 ml	[8,39,41–43]
*h*	*K* fraction Hill constant	7	3–9	[8,39,41–43]
*K*_min_	minimum *K* fraction	0	0	this study
*K*_max_	maximum *K* fraction	0.5	0.3–0.8	[8,39,41–43]
*K*_1/2_	*K* half-time constant	225 days	100–300 days	[8,39,41–43]
*γ*	decay rate of GFAP in blood	0.7 day^−1^	0.5–1 day^−1^	[41]
*C_H_*	baseline concentration in healthy individuals	0.012 ng ml^−1^	0–0.11 ng ml^−1^	[9]
*Q_N_*	average quantity of GFAP per cell	3.1 × 10^−4^ ng cell^−1^	1.3–5.7 × 10^−4^ ng cell^−1^	this study

To assess the impact heterogeneity and parameter changes have on detection, we ran sensitivity analyses. A combination of local and global sensitivity analyses was performed depending on the parameters. Local sensitivity analysis involves varying one parameter while average values are assumed for the remaining parameters. This has the limitation of only exploring first-order effects of the parameters on the model outcome. There are limited experimental data on certain parameters in our model and some of our model parameters are inextricably linked. This led us to run certain parameter combinations globally as discussed below. In the future, given further experimental data it would be possible to also use additional techniques for global sensitivity analysis of all the parameters and further explore the relationship between parameters and their impact on detection.

The parameters defining Gompertzian growth in GBMs were obtained from *in vivo* data of 106 untreated glioblastomas [14]. The heterogeneity in these parameters and ranges have been quantified and explored in this initial study and also in further studies [14,18]. Previously reported average densities of cells in non-necrotic (5714 × 10^4^ cells ml^−1^) and necrotic (4800 × 10^4^ cells ml^−1^) regions of the tumour were used to convert between volumes and cell numbers [44,45]. We used the results of previous studies and simulations of tumour growth to parametrize necrosis in the tumour and relate it to tumour growth [40].

**Figure 3.**
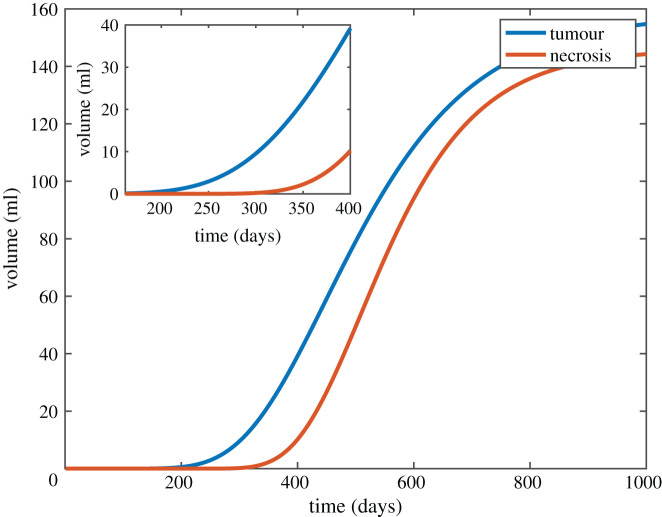
Gompertzian model of tumour and necrosis volumes as a function of time for average growth of untreated *in vivo* glioblastomas.

Figure 3 shows the average Gompertzian growth for GBMs. From the literature, we then took ranges for all the parameters which define tumour growth (*V_T_*_max_, *R_T_*), and for each set of these parameters, we derived an associated set of possible necrotic growth parameters (*V_N_*_max_, *R_N_*, *V_N_*_0_, *h*) from the range mentioned in the literature (table 1) within the constraints of *V_T_* > *V_N_*. As an example, electronic supplementary material, figure S3, shows the slowest, average and maximum tumour growth functions with all the corresponding possible necrotic growth functions. These constraints were used when running sensitivity analysis for the tumour and necrotic growth functions.

The parameters which govern the fraction of GFAP able to enter the bloodstream due to tumour growth (*K_T_*) are not well quantified (equation (2.5); *K*_max_, $K_{1/2}$, *h*). However, the processes involved, such as the perfusion (e.g. BBB permeability, blood flow), have been quantified for gliomas using a range of methods (e.g. dynamic perfusion CT and dynamic MRI) [42,43,46,47]. The relationship between these parameters and tumour volume has not been measured; we therefore fitted our parameters to match the available perfusion data along with data on the tumour volume at which biomarkers have been observed in the blood [6,9,10]. When running sensitivity analysis to determine the impact of these parameters, we note that the way we have fitted the parameters with the experimental data does not allow us to distinguish their individual effects. We therefore ran sensitivity analysis on all the possible parameter combinations for *K_T_* that match the experimental data simultaneously and assess these combined affects.

The remaining parameters (the patient's baseline healthy serum GFAP concentration (*C_H_*) [9] and the GFAP degradation rate (*γ*) [41]) are not dependent on the tumour but still affect the volume at which it is detected. To determine their combined effect, we ran sensitivity analysis simultaneously on these parameters for an average tumour (taking average values for all other parameters).

## 3. Results

### 3.1. *In silico* modelling of detection volume of glioblastoma multi-formes using serum glial fibrillary acidic protein

We model the dynamic changes in serum GFAP observed with tumour growth for a ‘typical’ GBM. This was achieved by using population averages to parametrize the model. This allowed us to obtain average values, for example, of detection volume. However, we note that GBMs are incredibly heterogenous which we examine in the next section. We combine previously published average parameters with our own experimental data (table 1) (see Material and methods for a further discussion of model parametrization).

The quantity of GFAP in a single cell (*Q_N_*) was determined *in* *vitro*. We found that the average mass of GFAP per G144 glioma stem cell was 1.7 × 10^−4^ ± 0.3 × 10^−4^ ng and per U251 GBM cell was 3.1 × 10^−4^ ± 0.8 × 10^−4^ ng.

**Figure 4.**
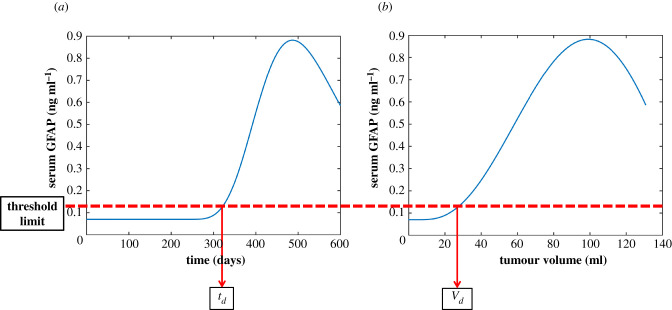
The serum GFAP concentration as a function of time (*a*) and tumour volume (*b*) was simulated for an average GBM. The panels show the serum GFAP concentrations for a GBM parametrized with average values and the threshold detection cut-off limit of 0.12 ng ml^−1^ (red) along with the corresponding detection time (*t_d_*) and detection volume (*V_d_*).

Figure 4 shows the dynamic change in serum GFAP as a function of time and tumour volume using population averages for all parameters in our model. The currently suggested threshold limit (0.12 ng ml^−1^) and corresponding detection tumour detection time (*t_d_*) and volume (*V_d_*) are also shown. The levels of GFAP only start to increase above the baseline level of healthy patients after the onset of necrosis and after the fraction entering the blood increases. Therefore, the tumour volume at which these changes occur (Vn0 and Vk0) is the crucial hard detection limit. The assay detection limit and heterogeneity across different patients will then determine the actual cut-off limit.

For a typical GBM with average parameter values, figure 4 can be used to convert GFAP serum concentration into tumour volume. Our model predicts that on average the serum GFAP would cross the currently suggested critical detection threshold for GBMs (0.12 ng ml^−1^ [5,9]) at a volume of 26 ml. If we lowered the threshold to the current analytical sensitivity and suggested a critical threshold for all glioblastomas (0.08 ng ml^−1^ [9]), we find that on average the tumour would be detected at a volume of 17 ml. Previous studies have found a correlation between tumour volume and serum GFAP levels [3,5,9,10]. Using these experimental averages at the limit of 0.12 ng ml^−1^, the average tumour volume was 23 ml and at 0.08 ng ml^−1^, it was 14 ml. These experimental data have a limited sample size but were still in good agreement with our model's predictions on the average detection volume at both these detection limits.

**Figure 5.**
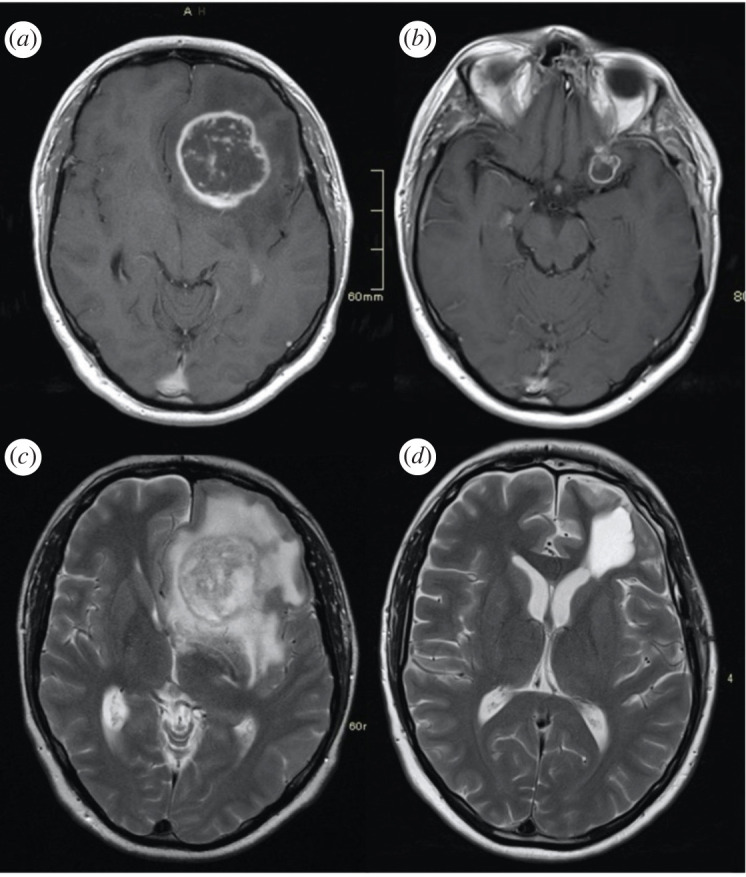
Representative images for a patient with remote recurrence of GBM in the contralateral hemisphere. T1-weighted post-gadolinium images (*a*,*b*) and T2-weighted images (*c*,*d*). (*a*) Pre-operative diagnostic imaging demonstrating a left frontal rim enhancing GBM with a significant volume of surrounding T2 hyperintensity. (*b*) Three months post-resection, post-adjuvant chemoradiotherapy (60 Gy in 30 fractions with concurrent temozolomide) baseline study.

To compare these average detection volumes with the volume at which GBMs are currently detected, the average GBM volumes for 449 patients at Leeds Teaching Hospitals NHS Trust were measured. Figure 5 shows representative images of a GBM patient.

Tumour size and volume were available for 449 patients—the median maximum enhancing tumour diameter was 4.2 cm (range 0.5–8.8 cm) and the median enhancing tumour volume was 23.6 ml (0.06–186 ml). Our results therefore suggest that using the critical cut-off threshold for GBMs (0.12 ng ml^−1^), serum GFAP cannot be used to detect GBMs earlier than current methods. However, by lowering this threshold, for example, to the current analytical sensitivity and suggested cut-off for glioblastomas (0.08 ng ml^−1^), it may be possible to use serum GFAP for early detection of GBMs.

Figure 4 shows how the levels of serum GFAP are predicted to rise with tumour growth except at the end of tumour growth. It has been shown that GBMs exhibit Gompertzian growth dynamics and that at larger tumour volumes, growth slows. As the maximum tumour and necrotic size is reached, the GFAP levels may decrease as the rate of necrosis slows. However, patient survival may prevent this limit ever being reached. Heterogeneity and, in some tumours, a decrease in GFAP expression with tumour growth have been observed in astrocytic tumours [48,49]. This causes a synonymous relationship between tumour volume and serum GFAP, which becomes increasingly heterogenous and, in some cases, lower with tumour growth [3,5,9].

### 3.2. Tumour heterogeneity and effect of key parameters on detection

There is large heterogeneity in the growth and behaviour of different GBMs, leading to analogous variations in the tumour detection volume (*V_D_*) at the threshold cut-off limit of 0.12 ng ml^−1^. For a given patient, the detection volume will depend on that patient's baseline parameters (*C_H_*, *γ*) and on the fundamental characteristics of that patient's tumour (all other model parameters).

**Figure 6.**
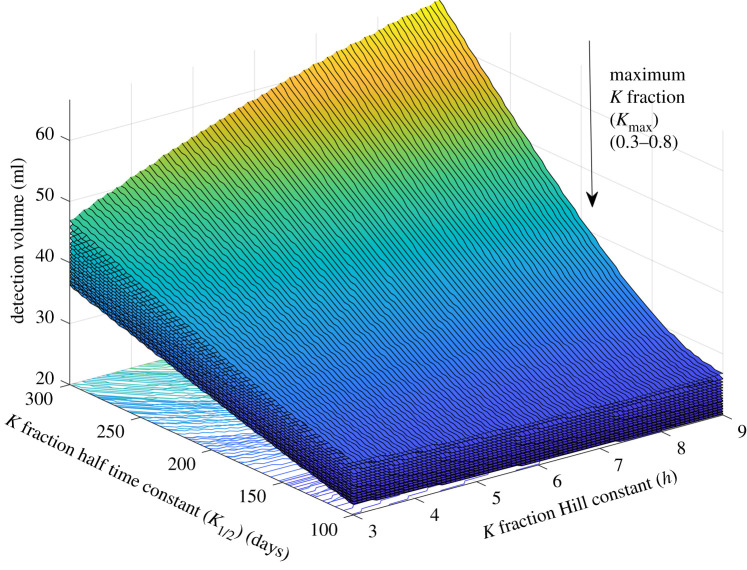
Volume that a tumour is detected (*V*_*D*_) for the possible combinations of parameters (*K*_1/2_, *h* and *K*_max_) which describe the function *K_T_* (the fraction of GFAP produced by the tumour that can enter the blood). *V_D_* is shown as a function of *K*_1/2_ and *h* for a range of *K*_max_ values (0.3–0.8). It was assumed for this sensitivity analysis that everything else about the patient and its tumour was average (average values assumed average for all other model parameters).

Sensitivity analysis can be used to examine the effect that changing a parameter will have on the detection volume (*V_D_*) and therefore assess its impact. Full descriptions of the different sensitivity analysis techniques performed can be found in the Material and methods. For the parameters defining the fraction of GFAP entering the blood $(K_{T})\;(\mathrm{equation}\;(2.5)$; $K_{\max},\;K_{1/2},\;h)$ sensitivity analysis was run on the parameter combinations that gave reasonable agreement with experimental data (figure 6). It was found that for average values of all other parameters, the detection volume (*V*_*D*_) across the possible (*K_T_*) parameter combinations varied from 20 to 58 ml giving a change in detection volume of $\Delta V_{D\;}=38\;\mathrm{ml}$.

**Figure 7.**
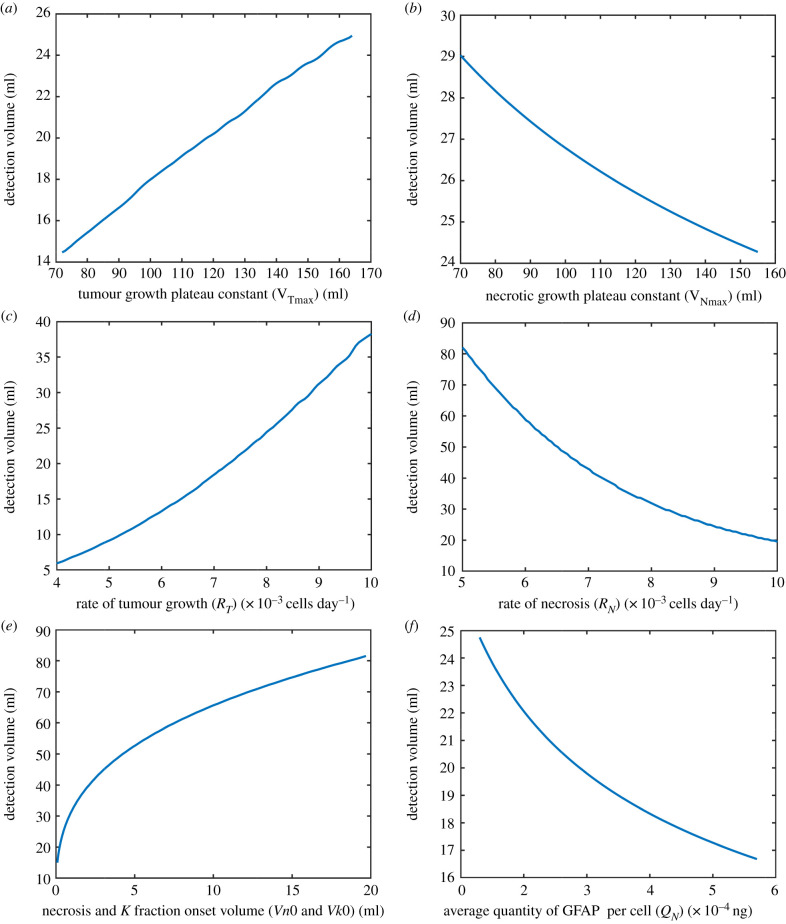
Sensitivity analyses showing the effect that the key parameters defining tumour growth, necrotic growth and the average quantity of GFAP per cell have on the volume at which a tumour is detectable (*V_d_*). The detection volume (*V_d_*) as a function of tumour (*V*_Tmax_) and necrosis (*V*_Nmax_) plateau constants (*a*,*b*), as a function of tumour growth rate (*R*_*T*_) and necrotic growth rate (*R_N_*) (*c*,*d*), as a function of necrosis and *K_T_* fraction onset tumour volume (*Vn*0 and *Vk*0) (*e*) and average quantity of GFAP per cell (*Q_N_*) (*f*).

Figure 7 shows how the remaining tumour-related parameters (those defining tumour growth (*V_T_*), necrotic growth (*V_N_*) and GFAP per cell (*Q_N_*)) influence the detection volume at the critical detection cut-off limit of 0.12 ng ml^−1^. The tumour and necrotic growth are closely related. In order to satisfy the condition that *V_T_* > *V_N_*, we only used corresponding tumour and necrotic growth parameter combinations (ranges listed in table 1) that satisfied this condition. The tumour detection volume (*V_d_*) is related to the ratio between tumour and necrotic growth. GFAP is produced as a consequence of necrotic growth not tumour growth and although the two are related it is possible to have a tumour which grows faster without having faster necrotic growth. As shown in figure 7*a*, if this occurs, the tumour will grow more before detection and be detected at a larger volume.

It can be seen in figure 7 that the rate of necrosis (*R_N_*) and the volume at which necrosis and the increase in the fraction entering the blood occur (Vn0 and Vk0) have the largest impact on the detection volume. The later the onset (larger Vn0 and Vk0) the larger the tumour will grow between this onset and detection. For example, a tumour with necrosis onset at 0.5 ml will grow 34 ml less before detection than a tumour which does not have necrosis until it is 50 ml in size. A tumour with rapid necrosis (*R*_N_ = 0.013 cells day^−1^) and average changes (Vn0 and Vk0=0.5 ml) will be detected at 19.5 ml, whereas a tumour with slow necrosis (*R*_*N*_ = 0.005) and late changes (Vn0 and Vk0=20 ml ) will be detected at 118 ml.

**Figure 8.**
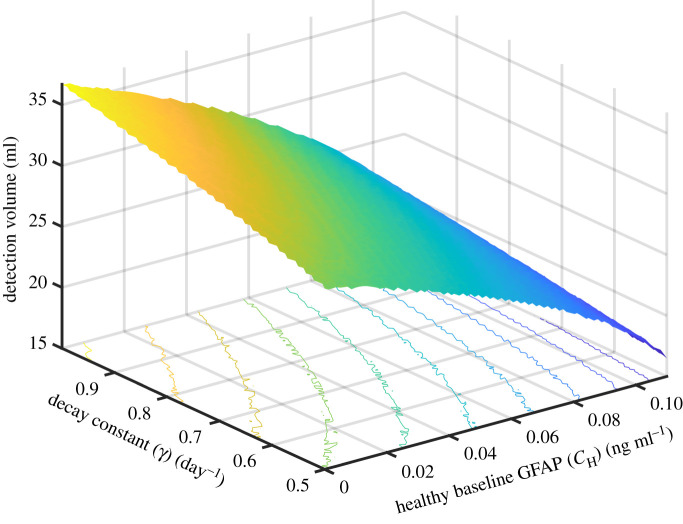
The tumour detection volume (*V*_D_) as a function of the patient's baseline healthy serum GFAP concentration (*C_H_*) and the degradation rate of GFAP (*γ*).

The patient's baseline healthy concentration (*C_H_*) and degradation rate (*γ*) are not tumour dependant but still affect the detection volume. Running simultaneous sensitivity analysis on both these parameters for an ‘average tumour’ (figure 8) we found that the combined effect of variations in both these parameters could be a change in detection volume of up to (ΔV_*d*_ = 20 ml). This means that baseline differences across patients could have a dramatic impact on detection. As seen in figure 8, these mainly stem from heterogeneity in the baseline concentration of GFAP in healthy patients. As the healthy baseline concentration of GFAP rises the GFAP contribution required from the tumour to reach the detection cut-off threshold drops and therefore the tumour is detected earlier at a smaller volume. To account for these differences and to detect tumours earlier, it may be possible to take dynamic measurements, by measuring the serum GFAP levels over time. This would allow a significant reduction in false positives and a significantly increase in the ROC. However, it should be noted that dynamic measurements will not improve false negatives in cases where tumours have low serum GFAP, due to low GFAP expression.

Tumours are incredibly heterogenous and are represented by a wide range of parameters, which leads to analogously heterogenous detection volumes and dynamic GFAP profiles. This could lead to larger errors if using population averages to convert the serum GFAP concentration to tumour volume. Previous studies have demonstrated, in line with the predictions of our model, that the serum concentration of GBMs of similar volumes can be extremely heterogenous [3,5,9,10]. For example, Kiviniemi *et al*. [3] saw a patient with a GBM volume of 15 ml who had a serum GFAP concentration of 0.4 ng ml^−1^ [3]. The GFAP heterogeneity observed experimentally is in good agreement with our results, but experimental data are limited and more data with more thorough quantification are required to fully quantify heterogeneity across different tumours.

Despite the large heterogeneity in the timescales involved in the dynamic GFAP concentration profiles (which depend on the characteristics of a specific tumour), for all tumours, the levels of GFAP initially follow the same trend, increasing with volume. Also, if a given tumour is not detectable until it is larger, this will be because the volumes associated with necrosis and the damage to the vasculature all occurred at larger volumes and/or that the rates of these were slower. The exception to this is tumours that exhibit very low GFAP expression. Therefore, even though serum GFAP is not always a reliable predictor of tumour volume, it is, beyond this exception, a good measure of BBB breakdown and necrosis. Both of which are key indicators of tumour severity [50–52].

### 3.3. Inputting and deriving additional clinical insight

**Figure 9.**
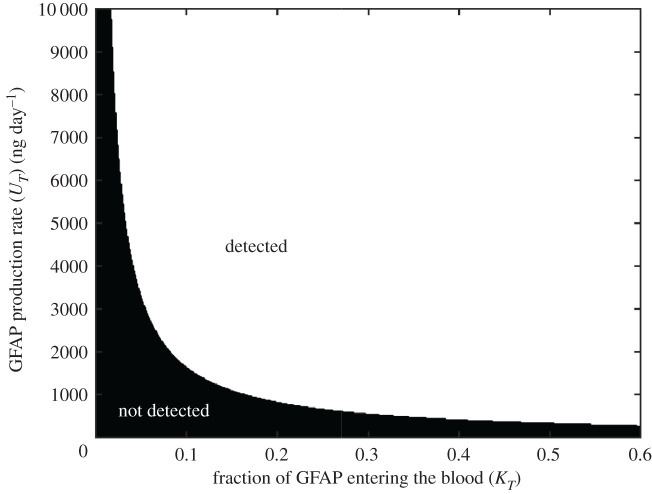
Combinations of GFAP production rate (*U_T_*) and fraction entering the blood (*K_T_*) result in a tumour being detected at the current detection limit of 0.12 ng ml^−1^.

Prospective studies on GFAP serum levels in brain tumour patients found that GFAP was detected in patients with other non-glial brain tumours [5,9,10]. This suggests that in addition to tumour necrosis, GFAP may also be produced because of astrocyte damage in the brain surrounding the tumour. Production via tumour necrosis occurs only in tumours derived from astrocytes, whereas external production may be induced by any type of brain tumour. Equation (2.2) can be more generally used to determine the combinations of *K_T_* and *U_T_* which give rise to serum GFAP levels above the current cut-off limit (figure 9).

Zero values of *U_T_* arise as a consequence of zero GFAP expression and/or no cell death, whereas the highest levels are represented by high levels of GFAP expression and rapid cell death. All other values in between these represent the other possible combinations of GFAP expression and cell death rates. If the BBB is not very severely compromised (*K_T_* < 0.1) then detection requires high production rates *U_T_* > 2000 ng day^−1^. Assuming an average quantity of GFAP per cell, this is equivalent to 3 × 10^6^ cells day^−1^ dying or a volumetric death rate of 0.04 ml day^−1^. Different parameter combinations can explain such a fast rate, but all combinations would require rapid death of a significant volume. On the other hand, if the BBB is severely compromised (*K_T_* = 0.4), then significantly lower production rates, *U_T_* > 700 ng day^−1^, are required for detection. This would be equivalent to 9 × 10^5^ cells day^−1^ dying or a volumetric death rate of 0.02 ml day^−1^. This equates to a far slower death of a much smaller volume. Previous studies [28,48] suggest that during GBM growth GFAP expression may decrease. This could impact detection as the cell death rate would have to increase to produce the same quantity of GFAP.

## 4. Discussion

We developed a novel *in silico* model to further understand the use of blood-based biomarkers for brain tumours. We focused on the putative biomarker GFAP and primarily on its use as a blood-based biomarker for GBMs.

We found that the relationship between serum GFAP kinetics and tumour growth strongly depends on the tumour. We used our model to assess the use of GFAP as a blood-based biomarker for the earlier detection of GBMs. We found that on average the currently suggested critical cut-off serum GFAP detection limit for GBMs of 0.12 ng ml^−1^ cannot be used for earlier detection of GBMs. However, for tumours with early onset of characteristics associated with poor prognosis (necrosis and vasculature damage), serum GFAP may be used for earlier detection. If the limit was lowered to the current analytical sensitivity and suggested critical threshold for all glioblastomas (0.08 ng ml^−1^), the average GBM would be detected at 17 ml—resulting in earlier detection. Lowering the detection limit comes at a cost to specificity; at the limit of 0.08 ng ml^−1^, a reduction in the specificity to other tumours was observed, but the specificity to healthy patients remains high (92.4%). This limit should therefore be explored further experimentally. As improvements are made to the testing of serum GFAP, this limit may be lowered and so earlier detection of GBMs using serum GFAP is likely.

There are three key requirements for elevated serum GFAP levels in brain tumour patients (electronic supplementary material, figure S4). First, GFAP must be present in affected cells. Second, there must be a mechanism for GFAP release (e.g. necrosis) and finally there must be a means for GFAP to pass into the blood. If not, then serum GFAP will not be detected. This sets a fundamental limit on early detection.

Our model predicted that in all cases where GFAP is detected at the current cut-off limit the patient would benefit from further diagnosis. In most cases, the GFAP levels scale with tumour severity and so several different cut-off limits may be implemented. These results match prospective experimental studies which have shown that GFAP levels scale with patient prognosis [3,9].

Our model predicts that the exception to GFAP levels scaling with tumour growth and severity occurs late in GBM growth when levels may begin to decrease. It has been shown previously that GFAP expression is influenced by astrocytoma grade [48]. GFAP positive cells are present in tumours of all malignancy grades with a tendency for decreased GFAP levels with increasing astrocytoma grade [9,48,53]. There is also a higher degree of heterogeneity in GFAP levels with increasing grade [5,9,48,53]. We measured average quantities of GFAP per cell experimentally and found, in line with these previous studies, that it was more variable in GBM cells compared to glioma stem cells. Some GBMs have even been shown to have very low levels of GFAP expression [48,49]. Our models predicted that the serum GFAP levels are synonymously heterogenous and, in some cases, lower at the later stages of GBM growth. This matches with the experimental data [3,9,10]. As our understanding, classification and quantification of different GBM types improve, it will be possible to incorporate changes in the GFAP expression with tumour growth for different tumour types into our models.

Our model was used to show that the two parameters which have the largest impact on the volume at which a specific GBM would be detectable are the rate of necrosis (*R_N_*) and the tumour volume at which necrosis first occurs and that the BBB begins to break down (*Vn*0/*Vk*0). The WHO has classified glial tumours according to characteristics, such as necrosis and vascular perfusion, as well as genetic features [2,50,54]. Our model suggests that GFAP levels may be a good measure of these, with levels indicative of necrosis, as well as vasculature damage. There is still a large variance in GBM patient outcomes driving the need for additional quantification metrics and further classification to allow more personalized therapies [54].

It may be possible to account for some patient heterogeneity and improve the accuracy of current assays by taking dynamic measurements. We have shown that parameters which are dependent on the patient rather than tumour characteristics, such as the patient's healthy baseline GFAP serum levels and the GFAP decay rate, also impact detection. Taking dynamic measurements could significantly improve the ROC allowing us to lower the critical detection limits. This could be especially powerful at the earlier stages of necrosis onset when the increase in necrosis is rapid and GFAP expression heterogeneity is lower [3,28,48]. These results may be used to inform the development of future assays. They may also be used to provide additional context to GFAP liquid biopsy results.

In the future, it may be possible to quantify heterogeneities and incorporate errors into our models and predictions for GFAP blood levels for different tumours. We have shown how mathematical modelling can be used to explore different scenarios. For example, we have shown how a specific patient's parameters may be used to predict best- and worst-case scenarios. Mathematical modelling can be combined with GFAP liquid biopsy results alongside other clinical data, such as scans, histological data and molecular profiling, to provide clinical insight. Integrating liquid biopsy with other clinical techniques could allow early detection and diagnosis for personalized precision treatment [55,56]. To be useful in the clinic, these must be combined in an accessible way. We suggest that decision trees at the point of care could offer an effective way to integrate all the information and aid clinicians in the diagnosis and in the development of personalized treatment plans [57–59].

We propose that serum GFAP levels may be especially useful in diagnosing early GBMs when used alongside other diagnostic methods. For example, our modelling showed that if imaging and histology fail to reveal necrosis, but the GFAP is very high, this may suggest several scenarios that clinicians need to consider. First, necrosis may have just begun, but be rapid. Second, the tumour may be causing peripheral brain damage resulting in GFAP release and damage to the BBB. Finally, there may be extensive vasculature damage, which could be confirmed, for example, via dynamic contrast-enhancing MRI [42,60,61].

It may also be possible to pair our models with data on tumour-induced brain deformation and damage [62,63] to assess the extent of tumour-induced brain damage. Tumour characteristics, e.g. nodular versus infiltrative, play a role in the solid-state stress exerted by a brain tumour [64]. Mathematical models have been developed to predict GBM progression based on a specific patient MRI data, demonstrating how these models may be integrated and used in the clinic [65]. We are always seeking to improve our classification of brain tumours and our results suggest that GFAP may help to differentiate between different tumours and characteristics.

In the future, with a better understanding of the processes which govern biomarker transport through brain tumours and into the bloodstream, it will be possible to incorporate tumour heterogeneity and transport dynamics into more complex and realistic models. To build a more accurate model, further quantification of processes, such as the breakdown of the BBB, would be required.

We have presented a generalized form of our model and framework to allow it to be adapted to any blood-based biomarker for a range of brain tumours. Our model describes how to build a model for any biomarker using information on biomarker production via the brain tumour and healthy tissue. The key model components for biomarker production via a brain tumour are tumour growth, biomarker production and the fraction of biomarker that can enter the bloodstream. These can be applied to any brain tumour, for any mechanisms of biomarker production using whichever models are most appropriate. The baseline level of serum biomarker can be obtained for any biomarker by measuring control groups. We have focused on GFAP as it is currently the serum biomarker with the most experimental data allowing model parametrization, but as more biomarkers and experimental data emerge, our model can be easily adapted and parametrized for a range of biomarkers.

We are at the early stages of determining how blood-based biomarkers may be used to diagnose and monitor brain tumours. We have shown how simple *in silico* models may be used to further understand the current limitations, uses and strategies for blood-based biomarkers for brain tumours. We have also shown how experimental work and clinical data can be used to enhance the model's relevance. The mathematical techniques we have developed could also be used more generally in the development and clinical interpretation of liquid biopsies for brain tumours.

## Data Availability

The scripts used in the study are openly accessible through https://github.com/JohannaABlee/serum-biomarkers-brain-tumours. The data are provided in the electronic supplementary material [66].
